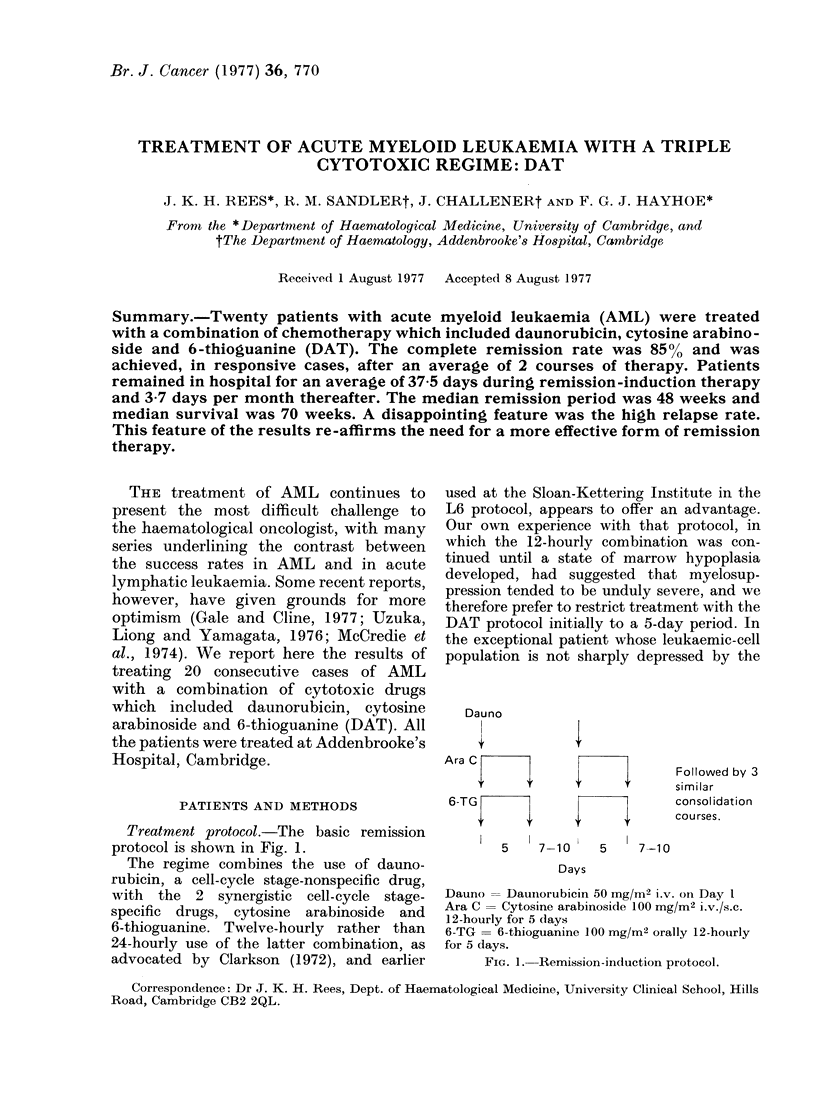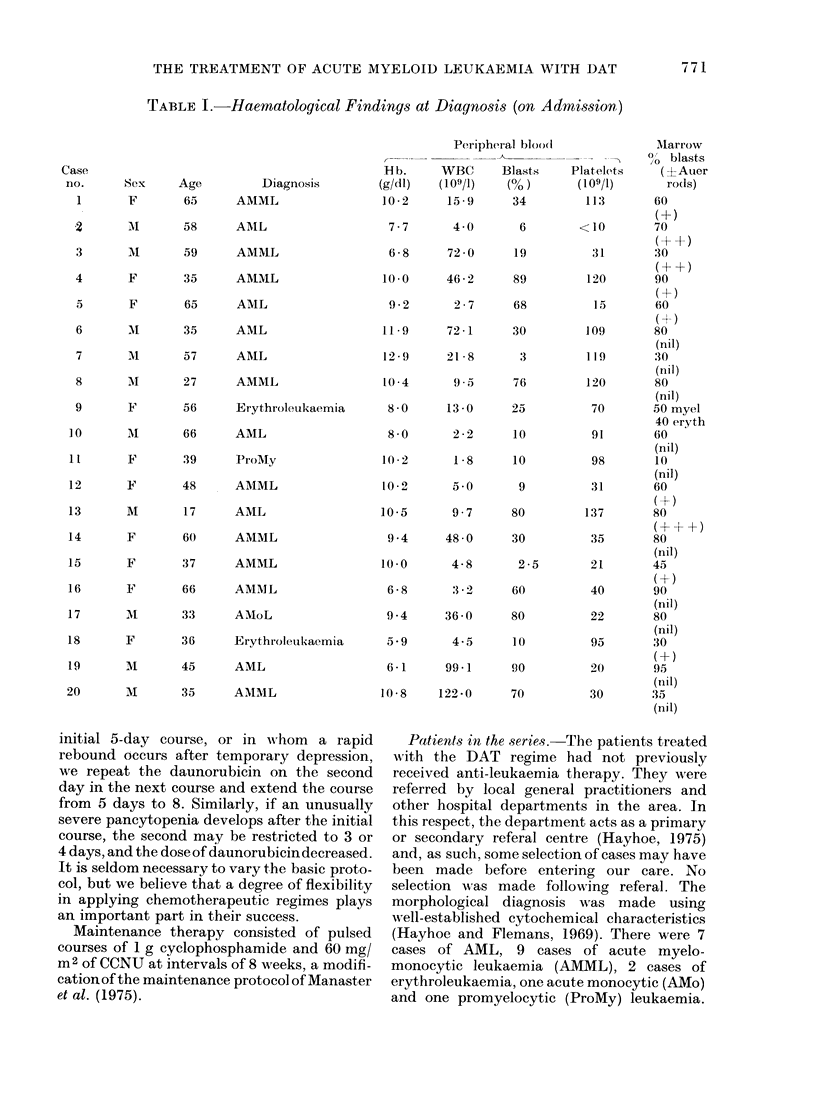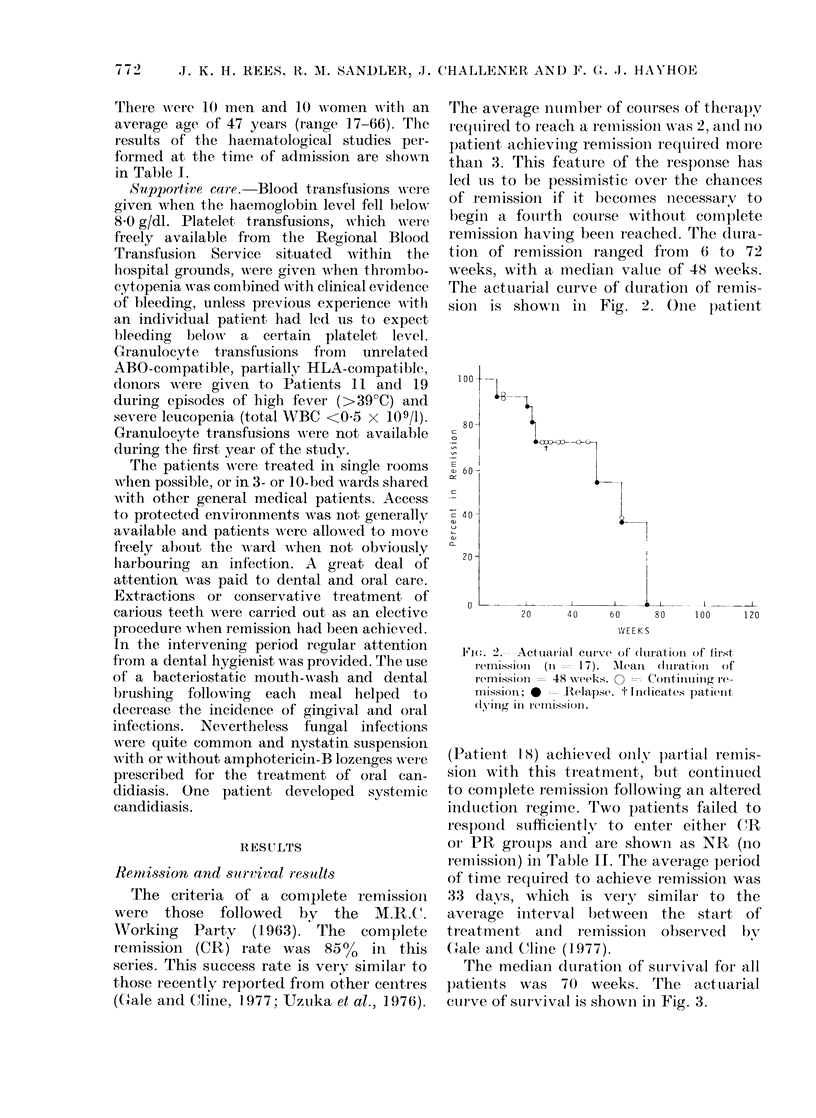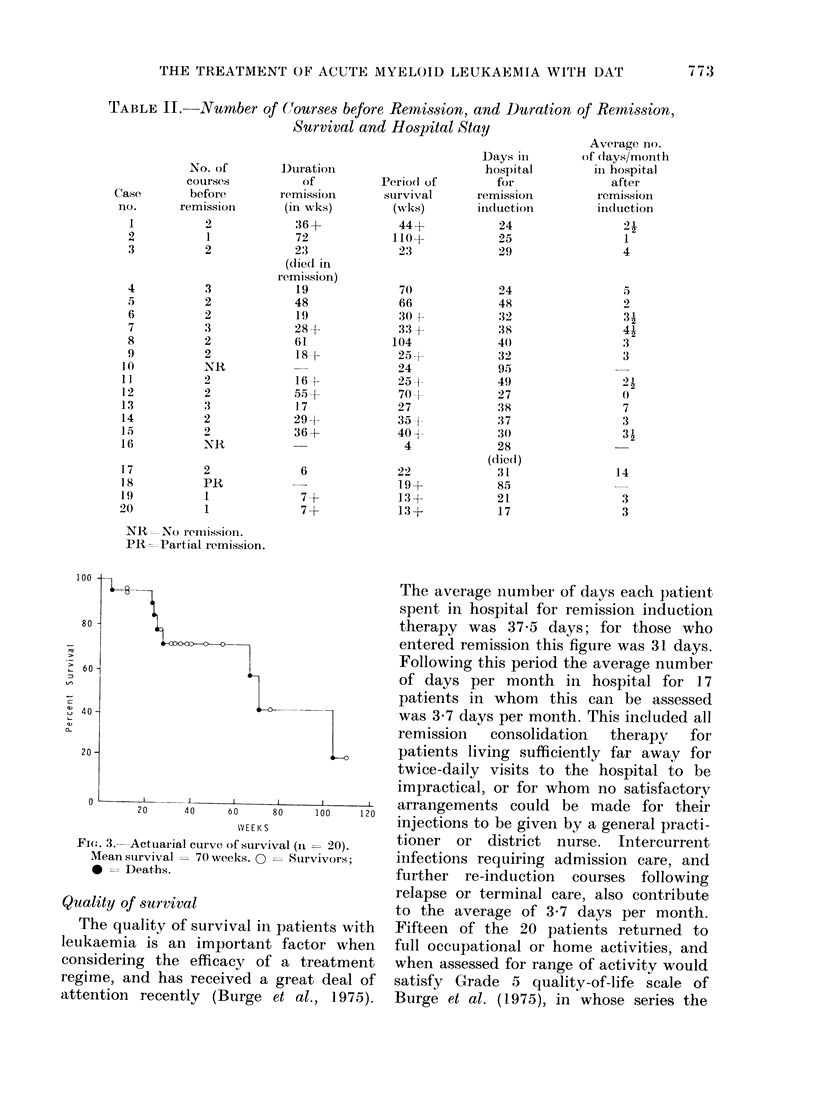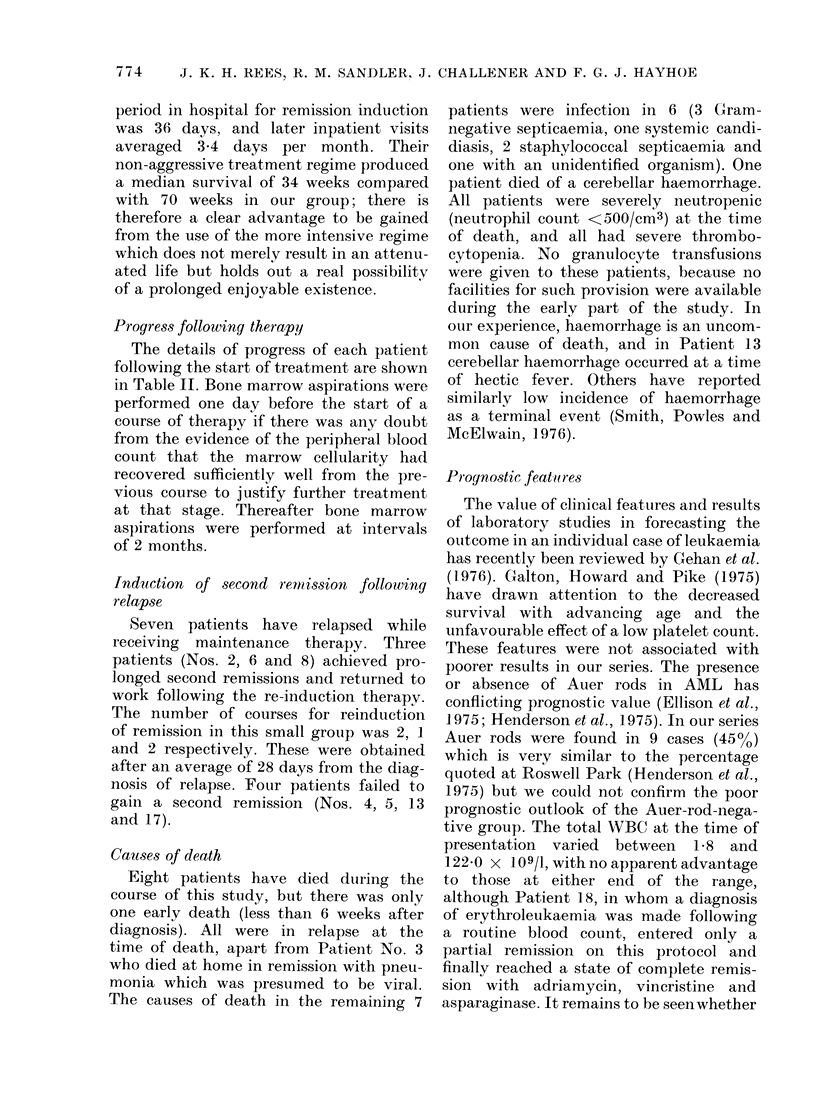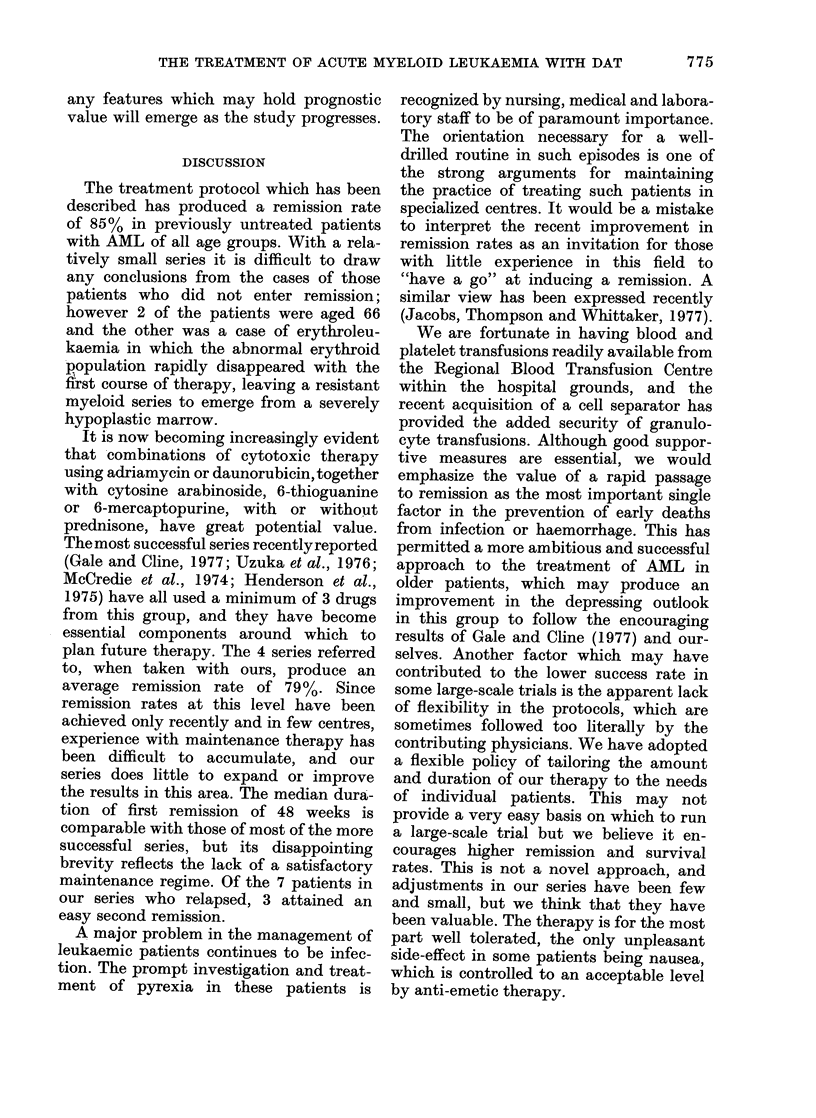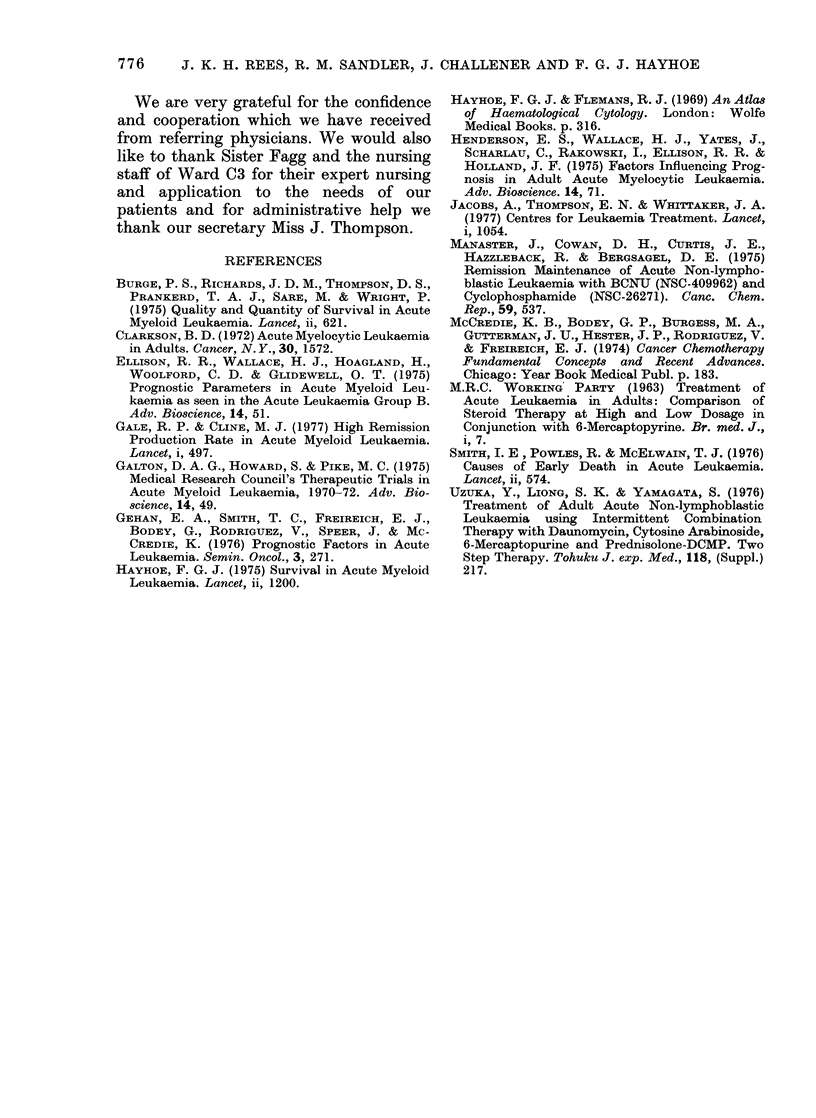# Treatment of acute myeloid leukaemia with a triple cytotoxic regime: DAT.

**DOI:** 10.1038/bjc.1977.260

**Published:** 1977-12

**Authors:** J. K. Rees, R. M. Sandler, J. Challener, F. G. Hayhoe

## Abstract

Twenty patients with acute myeloid leukaemia (AML) were treated with a combination of chemotherapy which included daunorubicin, cytosine arabino-side and 6-thioguanine (DAT). The complete remission rate was 85% and was achieved, in responsive cases, after an average of 2 courses of therapy. Patients remained in hospital for an average of 37.5 days during remission-induction therapy and 3.7 days per month thereafter. The median remission period was 48 weeks and median survival was 70 weeks. A disappointing feature was the high relapse rate. This feature of the results re-affirms the need for a more effective form of remission therapy.


*Br. J. Cancer* (1977) **36**, 770

### TREATMENT OF ACUTE MYELOID LEUKAEMIA WITH A TRIPLE CYTOTOXIC REGIME: DAT

J. K. H. REES*, R. M. SANDLER†, J. CHALLENER† AND F. G. J. HAYHOE*

*From the *Department of Haematological Medicine, University of Cambridge, and †The Department of Haematology, Addenbrooke's Hospital, Cambridge*

Received 1 August 1977 Accepted 8 August 1977

**Summary.—Twenty patients with acute myeloid leukaemia (AML) were treated with a combination of chemotherapy which included daunorubicin, cytosine arabinoside and 6-thioguanine (DAT). The complete remission rate was 85% and was achieved, in responsive cases, after an average of 2 courses of therapy. Patients remained in hospital for an average of 37·5 days during remission-induction therapy and 3·7 days per month thereafter. The median remission period was 48 weeks and median survival was 70 weeks. A disappointing feature was the high relapse rate. This feature of the results re-affirms the need for a more effective form of remission therapy.**

THE treatment of AML continues to present the most difficult challenge to the haematological oncologist, with many series underlining the contrast between the success rates in AML and in acute lymphatic leukaemia. Some recent reports, however, have given grounds for more optimism (Gale and Cline, 1977; Uzuka, Liong and Yamagata, 1976; McCredie *et al.*, 1974). We report here the results of treating 20 consecutive cases of AML with a combination of cytotoxic drugs which included daunorubicin, cytosine arabinoside and 6-thioguanine (DAT). All the patients were treated at Addenbrooke's Hospital, Cambridge.

### PATIENTS AND METHODS

*Treatment protocol.*—The basic remission protocol is shown in Fig. 1.

The regime combines the use of daunorubicin, a cell-cycle stage-nonspecific drug, with the 2 synergistic cell-cycle stage-specific drugs, cytosine arabinoside and 6-thioguanine. Twelve-hourly rather than 24-hourly use of the latter combination, as advocated by Clarkson (1972), and earlier used at the Sloan-Kettering Institute in the L6 protocol, appears to offer an advantage. Our own experience with that protocol, in which the 12-hourly combination was continued until a state of marrow hypoplasia developed, had suggested that myelosuppression tended to be unduly severe, and we therefore prefer to restrict treatment with the DAT protocol initially to a 5-day period. In the exceptional patient whose leukaemic-cell population is not sharply depressed by the

Dauno = Daunorubicin 50 mg/m² i.v. on Day 1
Ara C = Cytosine arabinoside 100 mg/m² i.v./s.c. 12-hourly for 5 days
6-TG = 6-thioguanine 100 mg/m² orally 12-hourly for 5 days.

FIG. 1.—Remission-induction protocol.

Correspondence: Dr J. K. H. Rees, Dept. of Haematological Medicine, University Clinical School, Hills Road, Cambridge CB2 2QL.

TABLE I.—*Haematological Findings at Diagnosis (on Admission)*

| Case no. | Sex | Age | Diagnosis | Peripheral blood Hb. (g/dl) | WBC ($10^9$/l) | Blasts (%) | Platelets ($10^9$/l) | Marrow % blasts (±Auer rods) |
|---|---|---|---|---|---|---|---|---|
| 1 | F | 65 | AMML | 10·2 | 15·9 | 34 | 113 | 60 (+) |
| 2 | M | 58 | AML | 7·7 | 4·0 | 6 | <10 | 70 (++) |
| 3 | M | 59 | AMML | 6·8 | 72·0 | 19 | 31 | 30 (++) |
| 4 | F | 35 | AMML | 10·0 | 46·2 | 89 | 120 | 90 (+) |
| 5 | F | 65 | AML | 9·2 | 2·7 | 68 | 15 | 60 (+) |
| 6 | M | 35 | AML | 11·9 | 72·1 | 30 | 109 | 80 (nil) |
| 7 | M | 57 | AML | 12·9 | 21·8 | 3 | 119 | 30 (nil) |
| 8 | M | 27 | AMML | 10·4 | 9·5 | 76 | 120 | 80 (nil) |
| 9 | F | 56 | Erythroleukaemia | 8·0 | 13·0 | 25 | 70 | 50 myel 40 eryth |
| 10 | M | 66 | AML | 8·0 | 2·2 | 10 | 91 | 60 (nil) |
| 11 | F | 39 | ProMy | 10·2 | 1·8 | 10 | 98 | 10 (nil) |
| 12 | F | 48 | AMML | 10·2 | 5·0 | 9 | 31 | 60 (+) |
| 13 | M | 17 | AML | 10·5 | 9·7 | 80 | 137 | 80 (+++) |
| 14 | F | 60 | AMML | 9·4 | 48·0 | 30 | 35 | 80 (nil) |
| 15 | F | 37 | AMML | 10·0 | 4·8 | 2·5 | 21 | 45 (+) |
| 16 | F | 66 | AMML | 6·8 | 3·2 | 60 | 40 | 90 (nil) |
| 17 | M | 33 | AMoL | 9·4 | 36·0 | 80 | 22 | 80 (nil) |
| 18 | F | 36 | Erythroleukaemia | 5·9 | 4·5 | 10 | 95 | 30 (+) |
| 19 | M | 45 | AML | 6·1 | 99·1 | 90 | 20 | 95 (nil) |
| 20 | M | 35 | AMML | 10·8 | 122·0 | 70 | 30 | 35 (nil) |

initial 5-day course, or in whom a rapid rebound occurs after temporary depression, we repeat the daunorubicin on the second day in the next course and extend the course from 5 days to 8. Similarly, if an unusually severe pancytopenia develops after the initial course, the second may be restricted to 3 or 4 days, and the dose of daunorubicin decreased. It is seldom necessary to vary the basic protocol, but we believe that a degree of flexibility in applying chemotherapeutic regimes plays an important part in their success.

Maintenance therapy consisted of pulsed courses of 1 g cyclophosphamide and 60 mg/$m^2$ of CCNU at intervals of 8 weeks, a modification of the maintenance protocol of Manaster *et al.* (1975).

*Patients in the series.*—The patients treated with the DAT regime had not previously received anti-leukaemia therapy. They were referred by local general practitioners and other hospital departments in the area. In this respect, the department acts as a primary or secondary referal centre (Hayhoe, 1975) and, as such, some selection of cases may have been made before entering our care. No selection was made following referal. The morphological diagnosis was made using well-established cytochemical characteristics (Hayhoe and Flemans, 1969). There were 7 cases of AML, 9 cases of acute myelomonocytic leukaemia (AMML), 2 cases of erythroleukaemia, one acute monocytic (AMo) and one promyelocytic (ProMy) leukaemia.

There were 10 men and 10 women with an average age of 47 years (range 17–66). The results of the haematological studies performed at the time of admission are shown in Table I.

*Supportive care.*—Blood transfusions were given when the haemoglobin level fell below 8·0 g/dl. Platelet transfusions, which were freely available from the Regional Blood Transfusion Service situated within the hospital grounds, were given when thrombocytopenia was combined with clinical evidence of bleeding, unless previous experience with an individual patient had led us to expect bleeding below a certain platelet level. Granulocyte transfusions from unrelated ABO-compatible, partially HLA-compatible, donors were given to Patients 11 and 19 during episodes of high fever (>39°C) and severe leucopenia (total WBC $<0{\cdot}5 \times 10^9$/l). Granulocyte transfusions were not available during the first year of the study.

The patients were treated in single rooms when possible, or in 3- or 10-bed wards shared with other general medical patients. Access to protected environments was not generally available and patients were allowed to move freely about the ward when not obviously harbouring an infection. A great deal of attention was paid to dental and oral care. Extractions or conservative treatment of carious teeth were carried out as an elective procedure when remission had been achieved. In the intervening period regular attention from a dental hygienist was provided. The use of a bacteriostatic mouth-wash and dental brushing following each meal helped to decrease the incidence of gingival and oral infections. Nevertheless fungal infections were quite common and nystatin suspension with or without amphotericin-B lozenges were prescribed for the treatment of oral candidiasis. One patient developed systemic candidiasis.

RESULTS

*Remission and survival results*

The criteria of a complete remission were those followed by the M.R.C. Working Party (1963). The complete remission (CR) rate was 85% in this series. This success rate is very similar to those recently reported from other centres (Gale and Cline, 1977; Uzuka *et al.*, 1976). The average number of courses of therapy required to reach a remission was 2, and no patient achieving remission required more than 3. This feature of the response has led us to be pessimistic over the chances of remission if it becomes necessary to begin a fourth course without complete remission having been reached. The duration of remission ranged from 6 to 72 weeks, with a median value of 48 weeks. The actuarial curve of duration of remission is shown in Fig. 2. One patient

FIG. 2.—Actuarial curve of duration of first remission (n = 17). Mean duration of remission = 48 weeks. ○ = Continuing remission; ● = Relapse. † Indicates patient dying in remission.

(Patient 18) achieved only partial remission with this treatment, but continued to complete remission following an altered induction regime. Two patients failed to respond sufficiently to enter either CR or PR groups and are shown as NR (no remission) in Table II. The average period of time required to achieve remission was 33 days, which is very similar to the average interval between the start of treatment and remission observed by Gale and Cline (1977).

The median duration of survival for all patients was 70 weeks. The actuarial curve of survival is shown in Fig. 3.

TABLE II.—*Number of Courses before Remission, and Duration of Remission, Survival and Hospital Stay*

| Case no. | No. of courses before remission | Duration of remission (in wks) | Period of survival (wks) | Days in hospital for remission induction | Average no. of days/month in hospital after remission induction |
|---|---|---|---|---|---|
| 1 | 2 | 36+ | 44+ | 24 | 2½ |
| 2 | 1 | 72 | 110+ | 25 | 1 |
| 3 | 2 | 23 (died in remission) | 23 | 29 | 4 |
| 4 | 3 | 19 | 70 | 24 | 5 |
| 5 | 2 | 48 | 66 | 48 | 2 |
| 6 | 2 | 19 | 30+ | 32 | 3½ |
| 7 | 3 | 28+ | 33+ | 38 | 4½ |
| 8 | 2 | 61 | 104 | 40 | 3 |
| 9 | 2 | 18+ | 25+ | 32 | 3 |
| 10 | NR | — | 24 | 95 | — |
| 11 | 2 | 16+ | 25+ | 49 | 2½ |
| 12 | 2 | 55+ | 70+ | 27 | 0 |
| 13 | 3 | 17 | 27 | 38 | 7 |
| 14 | 2 | 29+ | 35+ | 37 | 3 |
| 15 | 2 | 36+ | 40+ | 30 | 3½ |
| 16 | NR | — | 4 | 28 (died) | — |
| 17 | 2 | 6 | 22 | 31 | 14 |
| 18 | PR | — | 19+ | 85 | — |
| 19 | 1 | 7+ | 13+ | 21 | 3 |
| 20 | 1 | 7+ | 13+ | 17 | 3 |

NR = No remission.

PR = Partial remission.

FIG. 3.—Actuarial curve of survival (n = 20). Mean survival = 70 weeks. ○ = Survivors; ● = Deaths.

*Quality of survival*

The quality of survival in patients with leukaemia is an important factor when considering the efficacy of a treatment regime, and has received a great deal of attention recently (Burge *et al.*, 1975). The average number of days each patient spent in hospital for remission induction therapy was 37·5 days; for those who entered remission this figure was 31 days. Following this period the average number of days per month in hospital for 17 patients in whom this can be assessed was 3·7 days per month. This included all remission consolidation therapy for patients living sufficiently far away for twice-daily visits to the hospital to be impractical, or for whom no satisfactory arrangements could be made for their injections to be given by a general practitioner or district nurse. Intercurrent infections requiring admission care, and further re-induction courses following relapse or terminal care, also contribute to the average of 3·7 days per month. Fifteen of the 20 patients returned to full occupational or home activities, and when assessed for range of activity would satisfy Grade 5 quality-of-life scale of Burge *et al.* (1975), in whose series the period in hospital for remission induction was 36 days, and later inpatient visits averaged 3·4 days per month. Their non-aggressive treatment regime produced a median survival of 34 weeks compared with 70 weeks in our group; there is therefore a clear advantage to be gained from the use of the more intensive regime which does not merely result in an attenuated life but holds out a real possibility of a prolonged enjoyable existence.

*Progress following therapy*

The details of progress of each patient following the start of treatment are shown in Table II. Bone marrow aspirations were performed one day before the start of a course of therapy if there was any doubt from the evidence of the peripheral blood count that the marrow cellularity had recovered sufficiently well from the previous course to justify further treatment at that stage. Thereafter bone marrow aspirations were performed at intervals of 2 months.

*Induction of second remission following relapse*

Seven patients have relapsed while receiving maintenance therapy. Three patients (Nos. 2, 6 and 8) achieved prolonged second remissions and returned to work following the re-induction therapy. The number of courses for reinduction of remission in this small group was 2, 1 and 2 respectively. These were obtained after an average of 28 days from the diagnosis of relapse. Four patients failed to gain a second remission (Nos. 4, 5, 13 and 17).

*Causes of death*

Eight patients have died during the course of this study, but there was only one early death (less than 6 weeks after diagnosis). All were in relapse at the time of death, apart from Patient No. 3 who died at home in remission with pneumonia which was presumed to be viral. The causes of death in the remaining 7 patients were infection in 6 (3 Gram-negative septicaemia, one systemic candidiasis, 2 staphylococcal septicaemia and one with an unidentified organism). One patient died of a cerebellar haemorrhage. All patients were severely neutropenic (neutrophil count $<500/cm^3$) at the time of death, and all had severe thrombocytopenia. No granulocyte transfusions were given to these patients, because no facilities for such provision were available during the early part of the study. In our experience, haemorrhage is an uncommon cause of death, and in Patient 13 cerebellar haemorrhage occurred at a time of hectic fever. Others have reported similarly low incidence of haemorrhage as a terminal event (Smith, Powles and McElwain, 1976).

*Prognostic features*

The value of clinical features and results of laboratory studies in forecasting the outcome in an individual case of leukaemia has recently been reviewed by Gehan *et al.* (1976). Galton, Howard and Pike (1975) have drawn attention to the decreased survival with advancing age and the unfavourable effect of a low platelet count. These features were not associated with poorer results in our series. The presence or absence of Auer rods in AML has conflicting prognostic value (Ellison *et al.*, 1975; Henderson *et al.*, 1975). In our series Auer rods were found in 9 cases (45%) which is very similar to the percentage quoted at Roswell Park (Henderson *et al.*, 1975) but we could not confirm the poor prognostic outlook of the Auer-rod-negative group. The total WBC at the time of presentation varied between 1·8 and $122{\cdot}0 \times 10^9/l$, with no apparent advantage to those at either end of the range, although Patient 18, in whom a diagnosis of erythroleukaemia was made following a routine blood count, entered only a partial remission on this protocol and finally reached a state of complete remission with adriamycin, vincristine and asparaginase. It remains to be seen whether any features which may hold prognostic value will emerge as the study progresses.

### DISCUSSION

The treatment protocol which has been described has produced a remission rate of 85% in previously untreated patients with AML of all age groups. With a relatively small series it is difficult to draw any conclusions from the cases of those patients who did not enter remission; however 2 of the patients were aged 66 and the other was a case of erythroleukaemia in which the abnormal erythroid population rapidly disappeared with the first course of therapy, leaving a resistant myeloid series to emerge from a severely hypoplastic marrow.

It is now becoming increasingly evident that combinations of cytotoxic therapy using adriamycin or daunorubicin, together with cytosine arabinoside, 6-thioguanine or 6-mercaptopurine, with or without prednisone, have great potential value. The most successful series recently reported (Gale and Cline, 1977; Uzuka *et al.*, 1976; McCredie *et al.*, 1974; Henderson *et al.*, 1975) have all used a minimum of 3 drugs from this group, and they have become essential components around which to plan future therapy. The 4 series referred to, when taken with ours, produce an average remission rate of 79%. Since remission rates at this level have been achieved only recently and in few centres, experience with maintenance therapy has been difficult to accumulate, and our series does little to expand or improve the results in this area. The median duration of first remission of 48 weeks is comparable with those of most of the more successful series, but its disappointing brevity reflects the lack of a satisfactory maintenance regime. Of the 7 patients in our series who relapsed, 3 attained an easy second remission.

A major problem in the management of leukaemic patients continues to be infection. The prompt investigation and treatment of pyrexia in these patients is recognized by nursing, medical and laboratory staff to be of paramount importance. The orientation necessary for a well-drilled routine in such episodes is one of the strong arguments for maintaining the practice of treating such patients in specialized centres. It would be a mistake to interpret the recent improvement in remission rates as an invitation for those with little experience in this field to "have a go" at inducing a remission. A similar view has been expressed recently (Jacobs, Thompson and Whittaker, 1977).

We are fortunate in having blood and platelet transfusions readily available from the Regional Blood Transfusion Centre within the hospital grounds, and the recent acquisition of a cell separator has provided the added security of granulocyte transfusions. Although good supportive measures are essential, we would emphasize the value of a rapid passage to remission as the most important single factor in the prevention of early deaths from infection or haemorrhage. This has permitted a more ambitious and successful approach to the treatment of AML in older patients, which may produce an improvement in the depressing outlook in this group to follow the encouraging results of Gale and Cline (1977) and ourselves. Another factor which may have contributed to the lower success rate in some large-scale trials is the apparent lack of flexibility in the protocols, which are sometimes followed too literally by the contributing physicians. We have adopted a flexible policy of tailoring the amount and duration of our therapy to the needs of individual patients. This may not provide a very easy basis on which to run a large-scale trial but we believe it encourages higher remission and survival rates. This is not a novel approach, and adjustments in our series have been few and small, but we think that they have been valuable. The therapy is for the most part well tolerated, the only unpleasant side-effect in some patients being nausea, which is controlled to an acceptable level by anti-emetic therapy.

We are very grateful for the confidence and cooperation which we have received from referring physicians. We would also like to thank Sister Fagg and the nursing staff of Ward C3 for their expert nursing and application to the needs of our patients and for administrative help we thank our secretary Miss J. Thompson.

### REFERENCES

BURGE, P. S., RICHARDS, J. D. M., THOMPSON, D. S., PRANKERD, T. A. J., SARE, M. & WRIGHT, P. (1975) Quality and Quantity of Survival in Acute Myeloid Leukaemia. *Lancet*, ii, 621.

CLARKSON, B. D. (1972) Acute Myelocytic Leukaemia in Adults. *Cancer, N.Y.*, **30**, 1572.

ELLISON, R. R., WALLACE, H. J., HOAGLAND, H., WOOLFORD, C. D. & GLIDEWELL, O. T. (1975) Prognostic Parameters in Acute Myeloid Leukaemia as seen in the Acute Leukaemia Group B. *Adv. Bioscience*, **14**, 51.

GALE, R. P. & CLINE, M. J. (1977) High Remission Production Rate in Acute Myeloid Leukaemia. *Lancet*, i, 497.

GALTON, D. A. G., HOWARD, S. & PIKE, M. C. (1975) Medical Research Council's Therapeutic Trials in Acute Myeloid Leukaemia, 1970–72. *Adv. Bioscience*, **14**, 49.

GEHAN, E. A., SMITH, T. C., FREIREICH, E. J., BODEY, G., RODRIGUEZ, V., SPEER, J. & MCCREDIE, K. (1976) Prognostic Factors in Acute Leukaemia. *Semin. Oncol.*, **3**, 271.

HAYHOE, F. G. J. (1975) Survival in Acute Myeloid Leukaemia. *Lancet*, ii, 1200.

HAYHOE, F. G. J. & FLEMANS, R. J. (1969) *An Atlas of Haematological Cytology.* London: Wolfe Medical Books. p. 316.

HENDERSON, E. S., WALLACE, H. J., YATES, J., SCHARLAU, C., RAKOWSKI, I., ELLISON, R. R. & HOLLAND, J. F. (1975) Factors Influencing Prognosis in Adult Acute Myelocytic Leukaemia. *Adv. Bioscience.* **14**, 71.

JACOBS, A., THOMPSON, E. N. & WHITTAKER, J. A. (1977) Centres for Leukaemia Treatment. *Lancet*, i, 1054.

MANASTER, J., COWAN, D. H., CURTIS, J. E., HAZZLEBACK, R. & BERGSAGEL, D. E. (1975) Remission Maintenance of Acute Non-lymphoblastic Leukaemia with BCNU (NSC-409962) and Cyclophosphamide (NSC-26271). *Canc. Chem. Rep.*, **59**, 537.

MCCREDIE, K. B., BODEY, G. P., BURGESS, M. A., GUTTERMAN, J. U., HESTER, J. P., RODRIGUEZ, V. & FREIREICH, E. J. (1974) *Cancer Chemotherapy Fundamental Concepts and Recent Advances.* Chicago: Year Book Medical Publ. p. 183.

M.R.C. WORKING PARTY (1963) Treatment of Acute Leukaemia in Adults: Comparison of Steroid Therapy at High and Low Dosage in Conjunction with 6-Mercaptopyrine. *Br. med. J.*, i, 7.

SMITH, I. E , POWLES, R. & MCELWAIN, T. J. (1976) Causes of Early Death in Acute Leukaemia. *Lancet*, ii, 574.

UZUKA, Y., LIONG, S. K. & YAMAGATA, S. (1976) Treatment of Adult Acute Non-lymphoblastic Leukaemia using Intermittent Combination Therapy with Daunomycin, Cytosine Arabinoside, 6-Mercaptopurine and Prednisolone-DCMP. Two Step Therapy. *Tohuku J. exp. Med.*, **118**, (Suppl.) 217.